# Quality of life in families of infants and toddlers with food allergies: A mixed‐methods study

**DOI:** 10.1111/pai.70453

**Published:** 2026-08-13

**Authors:** Eleftheria Panagiotou, Eleni Andreou, Nicolas Nicolaou, Stella A. Nicolaou

**Affiliations:** ^1^ Department of Life Sciences University of Nicosia Nicosia Cyprus; ^2^ Department of Basic and Clinical Sciences, University of Nicosia Medical School University of Nicosia Nicosia Cyprus

**Keywords:** early childhood, food allergy, mixed methods, quality of life

## Abstract

**Background:**

Food allergies (FA) are a growing public health concern affecting family wellbeing. While previous research has focused largely on school‐aged children, limited evidence exists regarding families of infants and toddlers. This study examined the quality of life (QoL) in families with children aged up to 3 years with food allergies in Cyprus and sought to identify modifiable factors that may inform strategies to improve family QoL.

**Methods:**

A sequential explanatory mixed‐methods design was used. Quantitative data were collected from mothers of 100 children with FA diagnosis by either a consultant allergist or consultant paediatrician using the Food Allergy Quality of Life Questionnaire—Parent Form, translated and culturally adapted into Greek. Semi‐structured interviews were conducted with 12 mothers and analysed using thematic analysis. Findings were integrated using a joint display.

**Results:**

Mean FAQLQ‐PF scores indicated mild–moderate quality‐of‐life impairment; however, notable burden was observed in domains related to fear of unfamiliar foods, dietary restriction and social participation. Moderate–high concern regarding accidental ingestion (76%) and severe reactions (77%) was common. Qualitative findings highlighted sustained parental vigilance, restructuring of daily routines around food safety and emotional strain associated with diagnostic uncertainty. Mothers also described variability in clinical guidance and limited societal awareness, particularly in childcare and public food environments. Integration revealed predominantly convergent findings. In one area of complementarity, qualitative data revealed extensive daily caregiving effort underlying low quantitative activity limitation scores. One area of silence was identified where a quantitative construct was not reflected in interview narratives, suggesting that structured and open‐ended methods capture different dimensions of the caregiving experience.

**Conclusion:**

In our sample, families of infants and toddlers with FA in Cyprus experience persistent emotional and practical challenges despite moderate QoL impairment. Parental stress is driven primarily by risk perception, social restrictions and variability in clinical guidance. Standardised early management and improved community awareness may enhance family adaptation during this critical period.

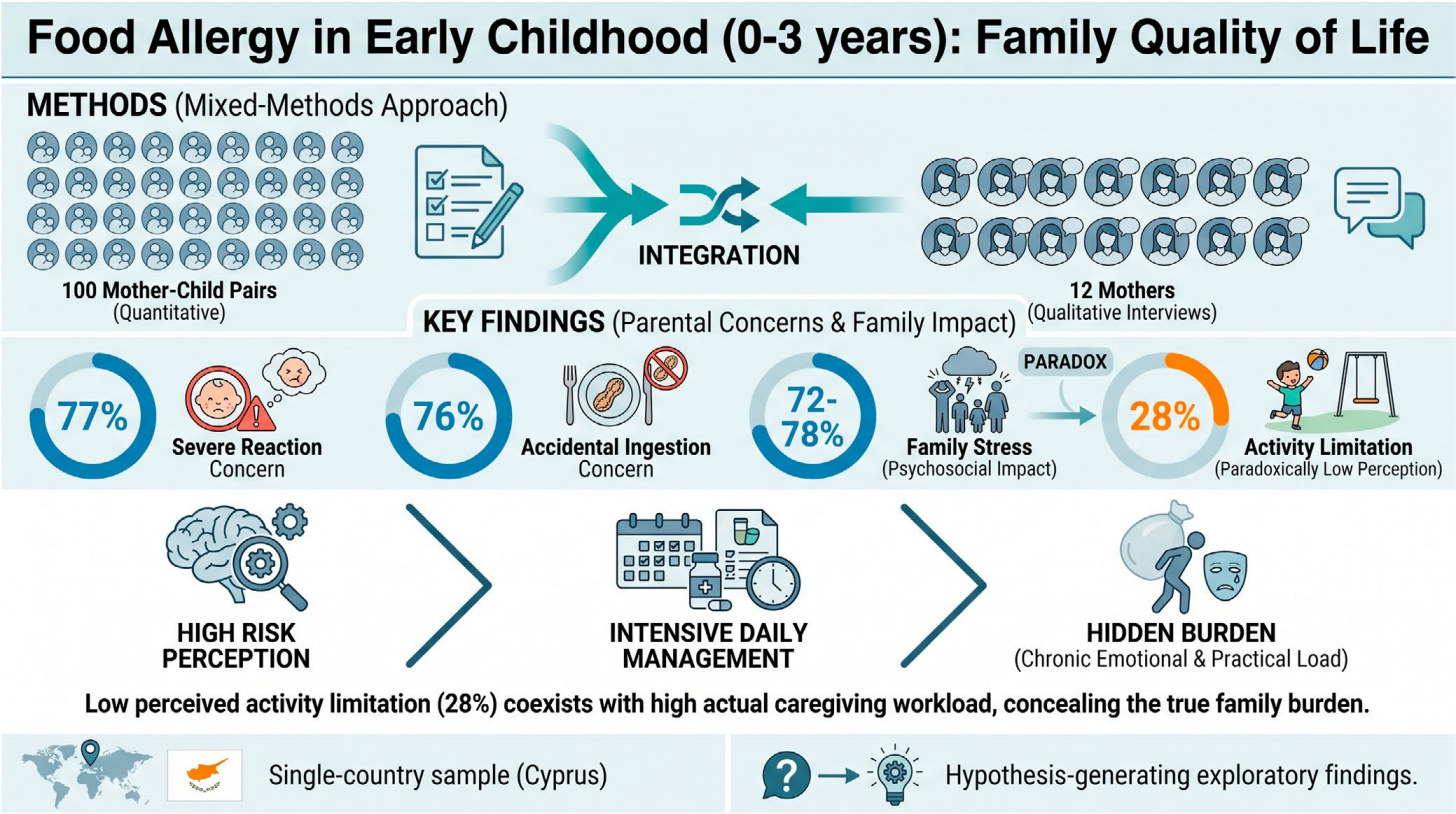

## Graphical abstract

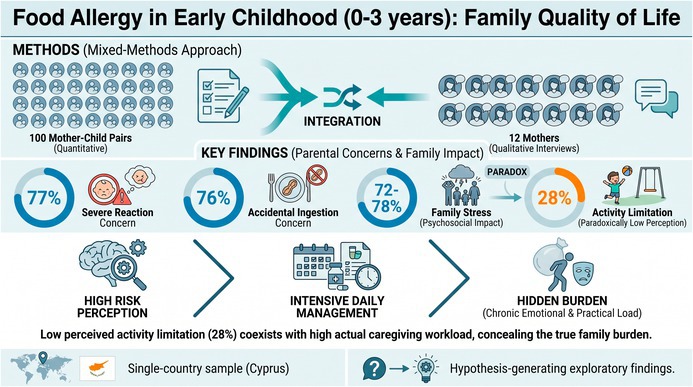

**Key message**

Food allergy in infants and toddlers is associated with significant family burden, highlighting the importance of structured early management and enhanced community awareness.

## 1 INTRODUCTION

Food allergy (FA) is among the most common chronic conditions of early childhood, affecting up to 10% of children worldwide, although prevalence estimates vary across regions and populations.^1^, ^2^, ^3^, ^4^, ^5^ Managing these allergies often extends beyond the medical aspect, affecting individuals' emotional wellbeing, social interactions and family daily routines.^6^, ^7^, ^8^, ^9^, ^10^

The first 3 years of life represent a critical developmental period during which immune maturation, gut microbiome development and dietary diversification occur simultaneously.^11^, ^12^, ^13^ When tolerance to dietary antigens does not develop appropriately, IgE‐mediated or non‐IgE‐mediated FA may arise.^13^

FA management in early childhood is largely caregiver‐driven, placing substantial responsibility on parents for allergen avoidance, symptom recognition and emergency preparedness. This responsibility can generate significant burden and anxiety, with parental perceptions of risk and disease severity strongly influencing family quality of life (QoL) and daily decision‐making.^7^, ^14^, ^15^ Families often respond through adaptive strategies such as heightened vigilance, environmental control and restructuring of food‐related routines to minimise exposure risk. However, most studies focus on school‐aged children, with less attention given to early childhood, when families are still adapting to management strategies.^7^, ^14^, ^15^

In Mediterranean societies such as Cyprus, food plays a central role in social interaction and family identity. Dietary restriction during early childhood may therefore carry additional social and emotional implications. Understanding the impact of FA within this cultural context is important for developing locally relevant clinical and educational interventions.^16^, ^17^

This study examined the impact of FA on family QoL among children aged up to 3 years in Cyprus and sought to identify modifiable factors that may inform strategies to support affected families. Using a sequential mixed‐methods design, it contributes to the literature by: (1) focusing on infancy and toddlerhood, a stage underrepresented in QoL research, (2) integrating quantitative FAQLQ‐PF outcomes with qualitative caregiving narratives and (3) examining these experiences within a Mediterranean sociocultural context.

## 2 METHODS

### 2.1 Participants

The quantitative analysis included data from 100 children diagnosed by either a consultant allergist or consultant paediatrician, as reported by their mothers. National demographic data indicate that there are approximately 10,000 live births annually in Cyprus, with an estimated FA prevalence of around 7% among infants and toddlers.^12^, ^18^ Sample size was estimated assuming a 95% confidence level, ±5% margin of error, 7% expected FA prevalence and a reference population of approximately 30,000 births across the relevant age range. In line with this calculation, a case sample of 100 mothers of children with clinically confirmed FA was recruited. This study is part of a larger project involving an additional 169 non‐allergic mother–child pairs. Comparative analyses between the allergic and non‐allergic groups will be reported in a future publication.

Eligible participants were mothers able to communicate in Greek or English with a child aged 3 years or younger. Data were collected between January 2024 and July 2025, and participants were recruited through allergist clinics, paediatric practices, nutrition services and social media platforms. The multi‐channel recruitment approach was adopted because the small target population in Cyprus made a single recruitment source insufficient to achieve the target sample size. Clinic‐based recruitment provided access to families with confirmed diagnoses and active clinical follow‐up, while social media extended reach to families not under ongoing specialist care.

All reported diagnoses were reviewed by a consultant allergist on the research team (NN), who assessed clinical plausibility based on symptom type, timing and suspected allergen, and classified each case as IgE‐mediated or non‐IgE‐mediated. Reactions with immediate cutaneous (e.g. urticaria, angioedema) or respiratory symptoms (e.g. cough, shortness of breath) within minutes to 2 h of exposure were classified as IgE‐mediated. Delayed reactions (>2 h after ingestion) presenting with gastrointestinal symptoms (e.g., persistent diarrhoea, blood in stools, multiple vomiting) or eczema flaring were classified as non‐IgE‐mediated FA cases. Specific diagnostic test data (e.g., SPT, specific IgE, oral food challenge) were not systematically collected for each participant. Among allergist‐diagnosed cases (63%), skin prick testing was the most commonly reported confirmatory method based on maternal report.

### 2.2 Food allergy quality of life—Parent form (FAQLQ‐PF)

The impact of a child's FA on family life was measured using the Food Allergy Quality of Life—Parent Form (FAQLQ‐PF), a validated 30‐item questionnaire for parents and caregivers covering emotional impact, food anxiety and social/dietary limitations.^19^

Items assessing child psychosocial and social–developmental domains are scored on a 0–6 Likert scale, parental stress and activity limitations on a 1–5 scale, and general health on a 0–6 scale. Additional categorical and frequency questions addressed FA management experiences. Scale scores were calculated as mean item responses, with higher scores indicating greater QoL impairment.

Internal consistency of the translated FAQLQ‐PF was excellent (*α* = 0.918), with strong reliability across subscales: child psychosocial impact (*α* = 0.934), social and developmental impact (*α* = 0.913), and parental risk and stress (*α* = 0.826). The three subscales align conceptually with the original three‐factor structure established by DunnGalvin et al., and items were assigned following the original instrument structure. Factor analysis was not conducted because the sample‐size‐to‐item ratio (3.3:1) falls below recommended minimum thresholds of 5:1–10:1.^19^, ^20^, ^21^ Test–retest reliability and convergent validity were not assessed as the FAQLQ‐PF was administered at a single time point and no second QoL instrument was included.^20^, ^22^

### 2.3 Translation and cultural adaptation

No validated Greek version of the FAQLQ‐PF (Parent Form) was available at study initiation. The instrument was translated and culturally adapted for Greek‐speaking parents in Cyprus where everyday Greek (Cypriot Greek) differs from Standard Modern Greek in vocabulary, register, idiomatic usage and the cultural connotations of specific terms. The translation followed the ISPOR Principles of Good Practice,^22^ with permission and guidance from the original developer (A. DunnGalvin). The process comprised two independent forward translations, reconciliation, two independent back‐translations by translators unfamiliar with the original, multidisciplinary expert panel review and pilot testing with eight parents of children with FA. To support the use of the adapted instrument in this study, internal consistency was confirmed (*α* = 0.918; subscales 0.826–0.934), and a significant positive correlation between the FAQLQ‐PF total score and the FAIM total score was observed (rho = 0.648, *p* < .001). These checks support within‐study use of the adapted instrument. A Greek FAQLQ‐PF was subsequently published by Vassilopoulou et al.; however, data collection for the present study had already commenced.^23^ A comprehensive psychometric evaluation, including item‐level comparison with the Vassilopoulou et al. version, is reported in a companion validation paper (Panagiotou et al., in preparation).

### 2.4 Semi‐structured interviews

Semi‐structured interviews were conducted to enable in‐depth exploration of maternal experiences of managing paediatric FA while allowing flexibility to capture individual perspectives. This qualitative phase complemented the quantitative findings by contextualising how FAQLQ‐PF domains were experienced in real‐world settings.

The interview guide was developed using preliminary FAQLQ‐PF findings, relevant qualitative literature and study objectives related to the development of a practical family support resource.^6^, ^9^, ^24^ Key domains included diagnostic experiences, everyday food management, dietary restriction, social participation, emotional responses, healthcare guidance and educational needs.

Twelve mothers who had completed the FAQLQ‐PF were purposively selected to capture variation in child age (4 months to 3 years), allergen type (milk protein, egg, nuts), region (Nicosia, Limassol) and time since diagnosis. Interviews were conducted face‐to‐face in Greek, lasted approximately 30 min and were audio‐recorded with informed consent. This sample size is consistent with evidence that thematic saturation in relatively homogeneous samples is typically achieved within 12 interviews.^25^ Saturation was monitored during data collection, defined as the absence of substantially new themes in later interviews and was reached at the tenth interview. The two additional interviews confirmed that no new themes emerged.

All interviews were conducted by EP, a doctoral researcher in nutrition and dietetics science with training in qualitative methods, who had no prior relationship with participants. Transcripts were independently coded by EP and SAN, with discrepancies resolved through discussion to consensus. The team's prior engagement with quantitative FAQLQ‐PF findings may have heightened sensitivity to burden‐related themes, which was managed through independent coding, attention to disconfirming cases and review of final themes by all authors. Formal member checking was not conducted and triangulation was achieved through mixed‐methods integration and independent coding with consensus discussion.

### 2.5 Data analysis

#### 2.5.1 Quantitative analysis

All quantitative data were analysed using IBM SPSS Statistics (version 28.0). Descriptive statistics were generated to summarise participant characteristics and QoL responses. Continuous variables were reported as means and standard deviations, and categorical variables were presented as frequencies and percentages. Percentages were rounded to the nearest whole number; therefore, totals may not sum exactly to 100%. Subgroup comparisons and multivariate analysis were considered but not conducted because of the sample size and the resulting subgroups with small numbers. The primary aim of the quantitative phase was to characterise QoL burden descriptively, with the qualitative phase providing explanatory depth. Comparative analyses between the FA and non‐allergic groups (*n* = 169) from the larger project will be reported separately.

#### 2.5.2 Qualitative analysis

Qualitative interview data were analysed using thematic analysis following the six‐phase framework of Braun and Clarke.^26^ Audio recordings were transcribed verbatim, and transcripts were analysed by EP and SAN. Familiarisation involved repeated reading and note‐taking, after which EP and SAN independently generated initial codes through line‐by‐line coding of all 12 transcripts. Codes were descriptive labels capturing discrete units of meaning (e.g., ‘carrying safe foods,’ ‘anxiety before new food introduction’). Through iterative comparison across transcripts, codes sharing a common thread were grouped into categories (e.g., ‘carrying safe foods,’ ‘home cooking,’ and ‘restaurant avoidance’ grouped under ‘environmental control outside the home’). Categories were reviewed for coherence and organised into candidate themes through constant comparison, then reviewed against the coded data, defined and named in line with study objectives. Illustrative quotations were retained to preserve participant voice.

A formal intercoder reliability coefficient was not calculated, consistent with reflexive thematic analysis.^26^ Coding was conducted manually using Microsoft Word.

Following separate quantitative and qualitative analyses, findings were integrated using a joint display approach guided by the frameworks of Fetters et al. and O’Cathain et al.^27^, ^28^ Data collection was sequential (quantitative findings informed the interview guide), but integration was iterative: qualitative themes were compared against quantitative domain scores, and emerging findings prompted re‐examination of specific quantitative items. The joint display (Table 1) paired each key quantitative finding with the most relevant qualitative theme, an illustrative quotation and an integrated interpretation. EP and SAN then examined whether qualitative data confirmed, extended, or complicated each quantitative finding and whether the combined interpretation yielded insights neither dataset would generate independently. All authors reviewed the integrated interpretations.

**TABLE 1:** Joint display integrating FAQLQ‐PF findings and qualitative interview themes.

FAQLQ‐PF (moderate‐high %)	Interview theme	Illustrative quote	Integration type	Integrated interpretation
78% family stress	Living with FA	‘Everything is affected‐the whole household is affected’	Convergence	High family stress is consistent with qualitative descriptions of household‐wide adaptation, in which FA reshapes daily routines and shared emotional dynamics
77% concern about severe reaction	FA management	‘We always carry antihistamines with us’	Convergence	High concern about severe reactions corresponds with qualitative accounts of emergency preparedness behaviours, including routine carrying of medication
76% concern about accidental ingestion	FA management	‘We need to know exactly what he is allergic to and inform others that it is dangerous for him and that no one should give it to him’	Convergence	Elevated risk perception is consistent with qualitative descriptions of proactive avoidance and communication strategies for allergen safety
75% moderate–high parental stress	Living with FA	‘Every time he eats something, I feel like I’m going to pass out’	Convergence	High parental stress aligns with qualitative accounts of sustained vigilance embedded in everyday caregiving routines
70% concern about child wellbeing	Living with FA	‘It makes me sad when my child has to miss out on foods she could have tried and enjoyed, like we do’	Convergence	Concern about child wellbeing corresponds with maternal expressions of sadness regarding perceived deprivation and missed dietary experiences
51% moderate‐high impact: ‘has to grow up more quickly than peers’	Not identified as a distinct qualitative theme	No spontaneous discussion across 12 interviews	Silence (quantitative without qualitative confirmation)	Over half of mothers endorsed this item quantitatively, yet the construct did not emerge in any interview despite coverage of developmental and social domains. This may indicate that the experience is difficult to articulate spontaneously or operates as an implicit assumption rather than a conscious concern
50% restricted environment	Educational and societal awareness	‘Situations like parties or eating outside the house make me really anxious because there's never complete control’	Convergence	Perceived environmental restriction is consistent with qualitative accounts of anxiety in settings where parental control over food is reduced
48% lack of dietary variety	Medical and diagnostic processes	‘We changed 2–3 types of formula until we found the right one’	Convergence	Lack of dietary variety is consistent with qualitative descriptions of trial‐and‐error feeding practices during and following the diagnostic process
46% afraid to try unfamiliar foods	Medical and diagnostic processes	‘Now, whenever I give him something new, I give him a very, very small amount to see if there is a reaction’	Convergence	Fear of unfamiliar foods corresponds with qualitative descriptions of cautious, stepwise food introduction following diagnosis
28% moderate‐high activity limitations	Living with FA	‘We always prepare food at home that he can eat, because you never know what will be available’, ‘We are fully prepared’	Complementarity	Low perceived activity limitation coexists with extensive qualitative accounts of daily preparation, including home cooking, carrying safe foods and restaurant avoidance. The FAQLQ‐PF captures whether families feel limited, but not the effort required to avoid limitation. This suggests that practical caregiving workload may be substantial even when perceived limitation is low

Each row was classified as convergence, complementarity, or silence following Fetters et al.^27^ Silence was identified when: (a) the finding had meaningful endorsement in the source dataset, (b) the other dataset provided adequate opportunity for the topic to surface and (c) systematic review of all 12 transcripts confirmed the absence of related codes. Where apparent discrepancies were observed, the team determined whether these represented complementarity or true contradiction (none identified). This study is reported in accordance with the Good Reporting of A Mixed Methods Study (GRAMMS) guidelines.^28^ A completed checklist is provided as Table S1.

### 2.6 Ethical approval

Ethical approval was granted by the Cyprus National Bioethics Committee (Reference: ΕΕΒΚ ΕΠ 2023.01.163). For the quantitative phase, all participants provided electronic informed consent via a secure online form prior to completing the FAQLQ‐PF. The consent form described the study purpose, procedures, voluntary nature of participation, data confidentiality and the right to withdraw at any time without consequence. For the qualitative phase, participating mothers provided additional written informed consent prior to each interview, including consent for audio recording. All data were stored in accordance with institutional data protection requirements.

## 3 RESULTS

### 3.1 FAQLQ‐PF findings

#### 3.1.1 Participant characteristics

A total of 100 children with FA were included (68% male; 47% aged 0–1 year, 30% aged 1–2 years, 23% aged 2–3 years). Most resided in urban areas (87%). More information is provided in Table 2. Regionally, children were primarily from Nicosia (48%) and Limassol (31%), with smaller representations from Larnaka (9%), Paphos (6%) and Famagusta (4%).

**TABLE 2:** Clinical and demographic characteristics of children with FA (*n* = 100).

Characteristic	%^a^ (*n*)
Sex	
Male	68 (68)
Female	31 (31)
No response	1 (1)
Age	
0–1 year	47 (47)
1–2 years	30 (30)
2–3 years	23 (23)
Residence	
Urban	87 (87)
Rural	11 (11)
No response	2 (2)
Region	
Nicosia	48 (48)
Limassol	31 (31)
Larnaka	9 (9)
Paphos	6 (6)
Famagusta	4 (4)
No response	2 (2)

^a^ Percentages calculated using available data.

#### 3.1.2 Food allergen categories and clinical characteristics of children with FA

Cow's milk protein was the most common allergen (44%), followed by egg (22%) and tree nuts (20%). Milk allergy predominated in infants aged 0–1 year (68%), while tree nut allergy increased with age. Most diagnoses were confirmed by consultant allergists (63%), and IgE‐mediated FA predominated (81%). Full details are presented in Table 3. Among IgE‐mediated cases (*n* = 81), skin manifestations were most common (96%), followed by gastrointestinal (15%), respiratory (6%) and circulatory symptoms (2%). Thirteen children (13%) had experienced anaphylaxis; of these, six (46%) were prescribed an epinephrine auto‐injector. All caregivers reported reassurance following prescription, although most (83%) also reported associated anxiety.

**TABLE 3:** Allergen profile by age and clinical characteristics of children with FA (*n* = 100).

Variable	0–1 yr.^c^ (*n* = 47)	1–2 yr.^c^ (*n* = 30)	2–3 yr.^c^ (*n* = 23)	Total %^c^ (*n*)
Allergen profile^a^				% (*n* = 100)
Milk protein	68% (32)	20% (6)	26% (6)	44% (44)
Egg	11% (5)	50% (15)	9% (2)	22% (22)
Peanut	0% (0)	10% (3)	17% (4)	7% (7)
Tree nuts^b^	13% (6)	23% (7)	30% (7)	20% (20)
Sesame/Tahini	9% (4)	7% (2)	13% (3)	9% (9)
Fruits (total)	4% (2)	7% (2)	13% (3)	7% (7)
Vegetables (total)	4% (2)	3% (1)	4% (1)	4% (4)
Coconut	0% (0)	0% (0)	4% (1)	1% (1)
Beef	0% (0)	3% (1)	0% (0)	1% (1)

Diagnosis confirmed by				% (*n* = 100)
Consultant allergist	55% (26)	77% (23)	61% (14)	63% (63)
Consultant paediatrician	45% (21)	23% (7)	39% (9)	37% (37)

Type of FA				% (*n* = 100)
IgE‐mediated	70% (33)	87% (26)	96% (22)	81% (81)
Non‐IgE‐mediated	30% (14)	13% (4)	4% (1)	19% (19)

Symptoms in IgE‐mediated FA^a^				% (*n* = 81)
Skin (itching, rash, hives, swelling)	68% (32)	80% (24)	96% (22)	96% (78)
Gastrointestinal (vomiting, diarrhoea, abdominal pain)	9% (4)	23% (7)	4% (1)	15% (12)
Respiratory (shortness of breath, throat tightness)	2% (1)	3% (1)	13% (3)	6% (5)
Circulatory (drowsiness, loss of consciousness)	0% (0)	3% (1)	4% (1)	2% (2)

Contact with other peer children with FA				% (*n* = 100)
Never	28% (13)	50% (15)	22% (5)	33% (33)
Rarely	60% (28)	47% (14)	74% (17)	59% (59)
Sometimes	11% (5)	3% (1)	4% (1)	7% (7)
Often	2% (1)	0% (0)	0% (0)	1% (1)

History of anaphylaxis				% (*n* = 100)
Ever had an anaphylactic reaction	13% (6)	10% (3)	17% (4)	13% (13)
No anaphylaxis	87% (41)	90% (27)	83% (19)	87% (87)

Timing of anaphylaxis				% (*n* = 13)
Very recent	6% (3)	3% (1)	0% (0)	30% (4)
6–12 months ago	9% (4)	3% (1)	9% (2)	54% (7)
1 year ago	0% (0)	0% (0)	9% (2)	15% (2)

Epinephrine auto‐injector prescription				% (*n* = 13)
Prescribed	4% (2)	3% (1)	13% (3)	46% (6)
Not prescribed	9% (4)	7% (2)	4% (1)	54% (7)

Impact of provision of EpiPen/Anapen (*n* = 6)				% (*n* = 6)
Caused reassurance	4% (2)	3% (1)	13% (3)	100% (6)
Caused anxiety	4% (2)	7% (2)	4% (1)	83% (5)
Did not cause anxiety	2% (1)	0% (0)	0% (0)	17% (1)

^a^ Participants may report more than one allergen or symptom.

^b^ Tree nuts include almonds, walnuts, cashews, pistachios, pecans, hazelnuts and macadamia nuts.

^c^ Percentages in age columns are calculated within age‐group denominators. Percentages in the Total column are calculated within the denominator specified for each section.

#### 3.1.3 FAQLQ—parent form

As shown in Table 4, child psychosocial impact was moderate‐to‐high in 34%–48% of respondents, with lack of dietary variety (48%), frustration with dietary restrictions (46%) and fear of unfamiliar foods (46%) the most frequently endorsed items. Social and developmental impact ranged from 35% to 51%, with the highest endorsement for having to grow up more quickly than peers (51%) and living in a more restricted environment (50%).

**TABLE 4:** Psychosocial, social, health and parental burden associated with childhood FA.

Category	Low %^a^ (*n*)	Moderate %^a^ (*n*)	High %^a^ (*n*)	Mean ± SD	Moderate‐High impact %^a^ (*n*)
Child psychosocial impact					
Feels worried about food (*n* = 98)	63% (62)	36% (35)	1% (1)	1.28 ± 1.23	37% (36)
Feels different from others (*n* = 98)	63% (62)	37% (36)	0% (0)	1.27 ± 1.10	37% (36)
Frustrated by dietary restrictions (*n* = 96)	54% (52)	43% (41)	3% (3)	1.49 ± 1.26	46% (44)
Afraid to try unfamiliar foods (*n* = 96)	54% (52)	43% (41)	3% (3)	1.61 ± 1.11	46% (44)
Worried about parent's concern (*n* = 96)	57% (55)	42% (40)	1% (1)	1.39 ± 1.09	43% (41)
Lacks dietary variety (*n* = 97)	52% (50)	46% (45)	2% (2)	1.66 ± 1.14	48% (47)
Experiences physical distress (*n* = 96)	63% (60)	35% (34)	2% (2)	1.43 ± 1.15	37% (36)
Experiences emotional distress (*n* = 96)	66% (63)	34% (33)	0% (0)	1.31 ± 0.98	34% (33)
Social and developmental impact					
Receives more attention than peers (*n* = 96)	51% (49)	44% (42)	5% (5)	1.82 ± 1.42	49% (47)
Has to grow up more quickly than other peers (*n* = 96)	49% (47)	48% (46)	3% (3)	1.75 ± 1.27	51% (49)
More restricted environment (*n* = 96)	50% (48)	46% (44)	4% (4)	1.74 ± 1.35	50% (48)
Restaurant limitations (*n* = 98)	55% (54)	43% (42)	2% (2)	1.79 ± 1.39	45% (44)
Holiday limitations (*n* = 98)	65% (64)	33% (32)	2% (2)	1.66 ± 1.37	35% (34)
Social activity limitations (*n* = 98)	63% (62)	36% (35)	1% (1)	1.67 ± 1.32	37% (36)
Parental risk perception					
Accidental ingestion risk of the allergen (*n* = 100)	24% (24)	61% (61)	15% (15)	2.77 ± 1.46	76% (76)
Severe reaction risk if ingested (*n* = 100)	23% (23)	67% (67)	10% (10)	2.67 ± 1.39	77% (77)
Risk of death from the allergy in the future (*n* = 100)	48% (48)	50% (50)	2% (2)	1.69 ± 1.02	52% (52)
Concern about treatment effectiveness (*n* = 100)	57% (57)	37% (37)	6% (6)	1.55 ± 1.20	43% (43)
General Health					
Parent's general health (*n* = 100)	0% (0)	43% (43)	57% (57)	4.52 ± 0.73	100% (100)
Child's general health (*n* = 100)	0% (0)	13% (13)	87% (87)	4.98 ± 0.77	100% (100)
Parental Concern					
Concern about child physical health (*n* = 100)	30% (30)	27% (27)	43% (43)	3.27 ± 1.15	70% (70)
Concern about child emotional wellbeing (*n* = 100)	30% (30)	26% (26)	44% (44)	3.28 ± 1.20	70% (70)
Parental stress and limitations					
Stress on parent (*n* = 100)	25% (25)	32% (32)	43% (43)	3.40 ± 1.15	75% (75)
Stress on partner (*n* = 100)	28% (28)	35% (35)	37% (37)	3.11 ± 1.12	72% (72)
Stress on family (*n* = 100)	22% (22)	40% (40)	38% (38)	3.23 ± 1.00	78% (78)
Family activity limitations (*n* = 98)	71% (70)	19% (19)	9% (9)	2.20 ± 0.84	28% (28)
Child activity limitations (*n* = 98)	71% (70)	21% (21)	7% (7)	2.15 ± 0.83	28% (28)

^a^ Percentages are calculated within each item and are presented as % (*n*). Total n varies slightly across items due to missing responses. For child psychosocial impact, social and developmental impact, parental risk perception and general health rating, items were rated on a 7‐point Likert scale (0–6), categorised as low (0–1), moderate (2–4) and high (5, 6). For parental concern and parental stress and limitations, items were rated on a 5‐point Likert scale (1–5), categorised as low (1, 2), moderate (3) and high (4, 5). Moderate–high impact represents the combined proportion of respondents reporting moderate or high levels. Mean values are reported as mean ± standard deviation (SD).

The most concentrated burden was observed in parental risk perception and stress. Moderate‐to‐high concern about accidental ingestion (76%) and severe reactions (77%) was reported by more than three‐quarters of parents, and 72%–78% reported moderate‐to‐high stress on themselves, their partner, or the family. In contrast, moderate‐to‐high activity limitations were reported by only 28%, representing the lowest‐burden domain. All parents rated their own and their child's general health as moderate‐to‐high, despite elevated psychosocial stress. Full item‐level data are presented in Table 4.

Descriptive statistics for FAQLQ‐PF subsections, total score and FAIM are shown in Table S3. All items were scored on a 0–6 scale, with higher scores indicating greater impairment or perceived risk.

The FAQLQ‐PF total score was 2.03 ± 0.69, indicating low‐to‐moderate impairment in QoL. Among subsections, emotional impact had the highest mean score (2.38 ± 0.75), followed by social and dietary limitations (1.79 ± 0.75) and food anxiety (1.73 ± 0.83). The FAIM mean score was 2.49 ± 0.49, reflecting moderate perceived risk. Spearman correlation showed a significant positive association between FAQLQ‐PF total score and FAIM (*ρ* = 0.648, *p* < .001), indicating that higher perceived risk was associated with poorer QoL.

### 3.2 Qualitative findings

Phase 2 comprised semi‐structured interviews with 12 mothers exploring experiences of managing paediatric FA. Participants were aged 26–39 years, with children aged 4 months to 3 years. Demographic details are presented in Table S4.

#### 3.2.1 Overview of themes

Thematic analysis identified four interrelated themes reflecting maternal experiences of managing paediatric FA in early childhood: (1) medical and diagnostic processes, (2) living with FA, (3) FA management and (4) educational and societal awareness. Together, these themes describe a progression from initial diagnostic uncertainty toward structured adaptation characterised by sustained vigilance and everyday risk management. A summary of themes and subthemes is presented in Table 5, with the complete coding framework provided in Table S2.

**TABLE 5:** Qualitative themes, subthemes, analytical interpretation and representative quotations.

Theme	Subthemes	Representative quote
Medical and diagnostic processes	Symptom recognition; Diagnostic and healthcare consultations; Specialist referral and allergy testing; Clinical monitoring and hospitalisation; Family allergy history	‘The paediatrician and the allergist explained the steps we needed to follow’
Living with FA	Home‐based food preparation and dietary avoidance; Careful food introduction; Environmental control within the household; Social participation challenges outside the home; Emotional and psychological responses	‘We always prepare food at home that he can eat, because you never know what will be available’
FA management	Medication use and avoidance strategies; emergency preparedness and epinephrine access; follow‐up monitoring and repeat testing; accidental exposure experiences; caregiving responsibility.	‘It is stressful, and I always have some level of worry’
Educational and societal awareness	Need for practical educational resources and guidance; Awareness in childcare and school settings; Variability in clinical guidance; Restaurant allergen transparency and labelling	‘Products should clearly indicate allergens ‐ for example, restaurant menus should be more detailed’

##### Theme 1: Medical and diagnostic processes

Diagnosis was described as a transition from uncertainty to structured management. Mothers reported an initial period of uncertainty and repeated healthcare consultations, with paediatricians typically serving as first contact and allergists providing diagnostic confirmation, most commonly through skin prick testing. Gastrointestinal symptoms, eczema and feeding concerns were perceived as difficult to interpret, contributing to anxiety prior to diagnosis. Specialist referral and follow‐up testing were experienced as reassuring and enhanced caregivers' sense of monitoring and control, as reflected in one mother's comment: ‘The paediatrician and the allergist explained the steps we needed to follow’. Family allergy history further influenced early vigilance and expectations regarding severity.

##### Theme 2: Living with FA

Allergen avoidance extended beyond dietary restriction to broader restructuring of everyday family routines. Routine activities, including eating outside the home, were frequently transformed into carefully planned and negotiated events reflecting limited trust in external food environments. Mothers described routinely carrying safe foods and medication, while the introduction of new foods during infancy was approached cautiously with gradual exposure and monitoring for reactions. Social contexts, including restaurants, family gatherings and childcare settings, were perceived as challenging due to reduced parental control and often required advance planning or restricted participation. As one participant noted, ‘We are very careful to bring our own food and do not eat from restaurants; we are fully prepared’.

##### Theme 3: FA management

Management of paediatric FA during early childhood was characterised by sustained anticipatory vigilance embedded in routine caregiving. Anxiety was described not only during acute reactions but also prior to meals, new food introductions and social situations involving potential exposure. As children developed awareness of dietary restrictions, emotional challenges emerged, including frustration and distress related to perceived differences from peers. Parents described balancing management of their child's emotional responses with regulation of their own anxiety. Although partner support mitigated aspects of the caregiving burden, responsibility for day‐to‐day management remained primarily maternal, with one mother stating, ‘A lot of anxiety, because the risk of accidentally consuming food with traces of milk protein is high’.

##### Theme 4: Educational and societal awareness

Mothers reported variability in post‐diagnostic guidance and frequently relied on experiential learning to navigate daily management. Safety within childcare settings and public food environments was perceived as dependent on individual awareness rather than standardised procedures, reinforcing parental responsibility beyond the home. Inconsistent allergen transparency in restaurants further contributed to uncertainty and heightened vigilance. These accounts suggest that gaps in institutional support shifted risk management toward caregivers in environments where control was limited. As one participant highlighted, ‘Restaurants or hotels should be clear and transparent, so you know which foods contain allergens’.

Representative quotes illustrating the analytical interpretation of each theme are presented in Table 5, while the complete list of quotes is provided in Table S2.

### 3.3 Integration of quantitative and qualitative findings

The qualitative interview guide was informed by preliminary FAQLQ‐PF findings to facilitate in‐depth exploration of domains demonstrating heightened burden, including parental stress, risk perception, dietary restriction and challenges in social participation. Interviews captured daily management practices, emotional responses to uncertainty, experiences of clinical guidance and safety perceptions across social and childcare contexts, complementing the quantitative data.

Integration of quantitative and qualitative data revealed strong convergence across key domains. Elevated parental stress and concerns regarding accidental ingestion identified in the FAQLQ‐PF were mirrored in interview narratives describing persistent anticipatory vigilance and environmental control strategies. Reported social and dietary limitations corresponded with qualitative accounts of restaurant avoidance, reliance on home‐prepared meals and ongoing negotiation within childcare settings. Maternal concern about child wellbeing was reflected in interview accounts of sadness regarding perceived deprivation and missed developmental experiences. Fear of unfamiliar foods, reported by 46%, corresponded with descriptions of cautious, stepwise food introduction practices following diagnosis. Overall, qualitative findings enriched interpretation of quantitative burden by illustrating how measured stress was embedded within routine caregiving practices and everyday risk management.

One area of apparent discrepancy was identified. Activity limitations received low quantitative endorsement (28% moderate‐to‐high), yet qualitative accounts described extensive daily restructuring. This was classified as complementarity: the FAQLQ‐PF captures whether families feel limited, while qualitative data reveal the effort required to avoid limitation. The integrated interpretation, that proactive strategies sustain low perceived limitation while concealing substantial caregiving workload, is an insight neither dataset generates alone. This interpretation derives from a single domain of the joint display (activity limitations) and is therefore exploratory. It is an important point that warrants future testing and at present does not provide a definitive conclusion.

One area of silence was also identified. The item ‘has to grow up more quickly than peers’ was endorsed by 51% of mothers, yet this construct did not emerge in any interview despite coverage of developmental, social and emotional domains. Systematic transcript review confirmed the absence of related codes. Following Fetters et al., this silence does not invalidate the quantitative finding but suggests the construct may be difficult to articulate spontaneously or may operate as an implicit assumption, highlighting the value of structured measures in capturing experiences not volunteered in open‐ended discussion.^27^

**FIGURE 1:**
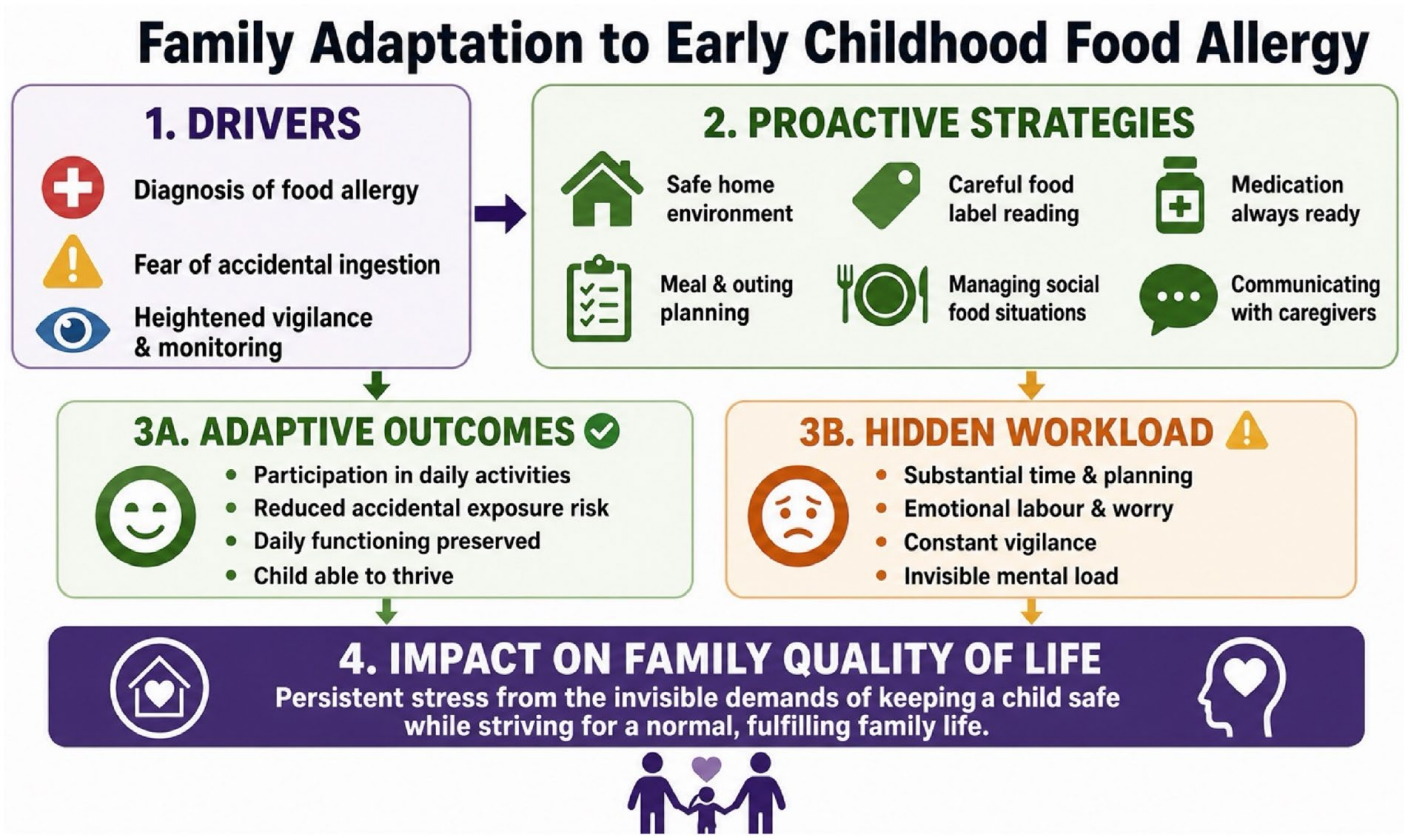
Proposed conceptual pathway linking parental risk perception, proactive adaptation and hidden caregiving burden in early childhood food allergy. High parental risk perception drives proactive management strategies that sustain low perceived activity limitation while generating a substantial caregiving workload not captured by standardised measures. The pathway is exploratory and hypothesis‐generating and further studies are warranted (Generated by Genspark AI).

As shown in Figure 1, diagnosis‐related increases in risk perception and vigilance drive proactive management strategies that support safe participation in daily life while simultaneously generating a substantial hidden caregiving workload that affects family QoL.

## 4 DISCUSSION

This mixed‐methods study examined the impact of FA on family QoL during early childhood in Cyprus. By integrating quantitative FAQLQ‐PF assessment with qualitative exploration, the study provides insight into family adaptation during the first 3 years of life of children with FA in Cyprus– a developmental period that remains underrepresented in QoL research. To our knowledge, this is the first mixed‐methods investigation focusing exclusively on families of infants and toddlers with FA in a Mediterranean context.

Overall, FAQLQ‐PF mean scores indicated mild‐to‐moderate impairment, consistent with findings in other paediatric FA QoL studies in early childhood. However, item‐level analysis revealed considerable heterogeneity across domains. Parental stress (72%–78% moderate‐to‐high), perceived risk of accidental ingestion (76%) and concern about severe reactions (77%) were substantially higher than activity limitations (28%). This pattern reflects the structure of multi‐domain QoL instruments: composite means aggregate across domains with varying burden levels, and high‐burden domains are attenuated by those with lower endorsement. Accordingly, the mild‐to‐moderate overall score should not be interpreted as indicating uniformly low burden. Rather, in this sample, the caregiving burden in early childhood FA is concentrated in risk perception, stress and social restriction, while perceived activity limitation remains comparatively low. These observations are descriptive; formal statistical comparisons between domains or subgroups were not conducted.

Parents rated both their own and their child's general health favourably, even while reporting high psychosocial stress. This coexistence of positive general health ratings and high domain‐specific stress has been reported in other FA populations and may reflect the distinct dimensions captured by general health items compared with allergy‐specific psychosocial measures.^6^, ^29^ Similar patterns of adaptation alongside persistent concern have been observed in previous studies.^10^, ^30^ In this sample, where children were aged 0–3 years and relied entirely on caregivers for food provision and safety monitoring, parental vigilance and emotional strain may be heightened relative to older age groups, though direct age‐group comparisons were beyond the scope of this study.^6^, ^10^, ^29^, ^30^

Quantitative data suggested that social and dietary limitations affected families as much as emotional distress. In the current sample, families frequently avoided restaurants, travel and social gatherings due to safety concerns, relying instead on home‐prepared foods and advance planning. While protective, these strategies reduced spontaneity and social participation, consistent with previous research.^7^, ^30^

Experiences with healthcare professionals varied widely, from clear communication to delays and limited guidance on emergency procedures. Similar variability in post‐diagnostic guidance has been reported in previous qualitative studies, which highlight unmet informational needs and inconsistencies in education regarding FA management and emergency preparedness.^6^, ^9^, ^29^, ^31^

Among the 13 children (13%) with parent‐reported anaphylaxis, fewer than half (46%; *n* = 6) had been prescribed an epinephrine auto‐injector, below the EAACI guidelines recommendation for auto‐injector prescription for all patients with prior anaphylaxis.^3^ While this finding may indicate gaps in emergency management or communication in early childhood FA care it should be interpreted with care, as several factors limit interpretation of this finding.^1^, ^32^ The subgroup is small (*n* = 13), anaphylaxis was identified through parental report and some episodes may not have met formal diagnostic criteria (e.g., WAO/EAACI grading). Data on clinical reasoning for prescribing decisions were not collected. This finding is hypothesis‐generating and should not be interpreted as evidence of guideline non‐adherence. Qualitative data provide supporting context as several mothers reported receiving no information about epinephrine auto‐injectors, suggesting that communication gaps and not necessarily inadequate care, may also contribute. Future studies with larger, clinically verified anaphylaxis cohorts should examine prescribing patterns, clinical decision‐making and communication about emergency management in early childhood FA care.

Limited public and institutional awareness of FA was frequently reported, with parents describing feeling unsupported in schools, restaurants and social environments. Participants highlighted the need for practical educational resources, including digital guidance and allergen‐specific food lists. Similar gaps have been reported in previous qualitative research.^24^, ^33^ Within the Cypriot context, improved allergen labelling in restaurants and packaged foods may further enhance safety and reduce parental anxiety, supporting greater inclusion of families in public food environments.

Integration revealed one area of complementarity with implications for measurement. Only 28% of mothers reported moderate‐to‐high activity limitations on the FAQLQ‐PF, yet qualitative accounts documented extensive daily caregiving effort, including routine home cooking, carrying safe foods to external settings and advance planning or avoidance of restaurants. We define this as ‘hidden burden’: caregiving effort that does not register on standardised activity limitation measures because proactive strategies prevent families from experiencing perceived limitation, while the workload sustaining this adaptation remains substantial. This term is used descriptively to characterise the observed complementarity rather than as a formally validated construct. Because this observation derives from a single domain of the joint display, it should be regarded as exploratory and hypothesis generating. Further studies are warranted prior to broader conceptual application. Still, the findings suggest that activity limitation scores in early childhood FA may warrant cautious interpretation alongside qualitative or behavioural data on daily management practices.

Several limitations should be acknowledged. FA diagnosis relied on parental report without verification against medical records. All cases were reviewed by a consultant allergist (NN) and 63% were allergist‐diagnosed; however, specific diagnostic test data were not systematically collected and formal misclassification assessment was not performed. Misclassification risk is greater for the non‐IgE‐mediated subgroup (19%), where symptoms overlap with non‐allergic conditions and any misclassification would most likely attenuate observed QoL effects. The sample included only mothers, reflecting their predominant caregiving role in early childhood FA management. Recruitment through allergy clinics and social media may favour more actively engaged parents, and the predominantly urban sample (87%) may underrepresent rural families or those less connected to specialist care. The findings should therefore be interpreted as reflecting the experiences of families actively managing their child's FA rather than all affected families in Cyprus. Taken together, reliance on parental report, diagnostic uncertainty and recruitment through clinics and social media may limit generalisability to less engaged or less health‐literate families, whose experiences and reported burden could differ from those captured here. The cross‐sectional design precludes assessment of longitudinal QoL changes, though it provides insight into early‐stage family adjustment following diagnosis. The complementarity finding regarding ‘hidden burden’ derives principally from a single domain of the joint display and should be regarded as exploratory. The study was not designed a priori to quantify the discrepancy between perceived limitation and caregiving effort. A dedicated design incorporating, for example, time‐use measures or behavioural logs alongside the FAQLQ‐PF would be required to establish this relationship more firmly.

The study translation includes deliberate cultural adaptations for the Cypriot Greek context alongside a small number of non‐culturally attributable differences. Strong internal consistency (*α* = 0.918) and concordance in item content, scale direction and endpoints support within‐study reliability; however, cross‐study score comparisons with the Vassilopoulou et al.^23^ Standard Modern Greek version should be interpreted with caution. Psychometric evaluation was limited to internal consistency. Factor analysis, test–retest reliability and convergent validity were not assessed due to sample size and the single‐administration design. FAQLQ‐PF scores reported here should therefore be interpreted as descriptive indicators of burden within this study rather than as values directly comparable to those from other validated language versions. Future research should incorporate objectively verified diagnoses, multi‐informant perspectives, longitudinal designs and larger samples for comprehensive psychometric evaluation, and cross‐cultural equivalence testing of the Greek Cypriot FAQLQ‐PF. Accordingly, the FAQLQ‐PF was used here as a culturally adapted study instrument rather than as a formally validated measure; comprehensive psychometric validation is reported separately.

## 5 CONCLUSION

In the current study, families of infants and toddlers with FA in Cyprus experience persistent emotional and practical challenges despite moderate QoL impairment. Parental stress is driven primarily by risk perception, social restrictions and variability in clinical guidance. Consistent with the study aim of identifying modifiable factors, our findings point to gaps in caregiver education on allergen avoidance and emergency management, inconsistent protocols in childcare and early education settings, and limited allergen transparency in food environments within the Cypriot context. Addressing these factors through structured early‐life FA support may reduce caregiver burden during this critical developmental period and improve family QoL by shifting part of the management responsibility from individual families to coordinated healthcare and community support structures.

## AUTHOR CONTRIBUTIONS

**Eleni Andreou:** Supervision; writing – review and editing; investigation; conceptualization; methodology. **Nicolas Nicolaou:** Conceptualization; investigation; writing – review and editing; supervision; data curation; methodology. **Eleftheria Panagiotou:** Conceptualization; investigation; writing – original draft; methodology; validation; visualization; formal analysis; data curation. **Stella A. Nicolaou:** Conceptualization; investigation; writing – original draft; writing – review and editing; methodology; visualization; formal analysis; project administration; supervision; data curation.

## CONFLICT OF INTEREST STATEMENT

The authors declare no conflicts of interest.

## Supporting information


**Table S1.** Good Reporting of A Mixed Methods Study (GRAMMS) checklist.

**Table S2.** Detailed coding framework including themes, categories, codes, participant contributions and illustrative quotations (Participant 1–12).

**Table S3.** Descriptive statistics of FAQLQ‐PF subscales, total score and FAIM total score (*n* = 100).

**Table S4.** Demographics of mother and children with FA.

## Data Availability

The data that supports the findings of this study are available in the supporting information S1 of this article.
