# Supplementary material for: Quality of life in families of infants and toddlers with food allergies: A mixed‐methods study
Source: Pediatr Allergy Immunol. 2026 Aug 13;37(8):e70453. doi: 10.1111/pai.70453 (PMC13473807; doi:10.1111/pai.70453)
Supplement: Supplementary file 1 [file PAI-37-e70453-s001.docx]

**Supplemental Table S3.** Descriptive statistics of FAQLQ-PF subscales, total score, and FAIM total score (n = 100)

| Variable | Mean ± SD | Median | Min | Max |
| --- | --- | --- | --- | --- |
| FAQLQ-PF Total Score | 2.03 ± 0.69 | 1.85 | 0.52 | 3.74 |
| Food Anxiety | 1.73 ± 0.83 | 1.63 | 0.00 | 3.88 |
| Emotional Impact | 2.38 ± 0.75 | 2.21 | 0.67 | 4.25 |
| Social & Dietary Limitations | 1.79 ± 0.75 | 1.57 | 0.29 | 3.43 |
| FAIM Total Score | 2.49 ± 0.49 | 2.50 | 1.33 | 4.17 |

*Questionnaire items were scored on a scale from 0 to 6, with higher scores indicating greater impairment or higher perceived risk (0 = lowest, 6 = highest). FAQLQ-PF = Food Allergy Quality of Life Questionnaire - Parent Form; FAIM = Food Allergy Independent Measure.
