# Supplementary material for: Quality of life in families of infants and toddlers with food allergies: A mixed‐methods study
Source: Pediatr Allergy Immunol. 2026 Aug 13;37(8):e70453. doi: 10.1111/pai.70453 (PMC13473807; doi:10.1111/pai.70453)
Supplement: Supplementary file 2 [file PAI-37-e70453-s002.docx]

**Supplementary Table S2:** Detailed coding framework including themes, categories, codes, participant contributions, and illustrative quotations (Participant 1-12)

| **Theme** | **Category** | **Code** | **Participants** | **Quotes** |
| --- | --- | --- | --- | --- |
| **1. Medical and Diagnostic Processes** | Diagnosis | Doctor (Pediatrician) | P1, P2, P3, P4, P5, P7, P9, P10, P11, P12 | P1 - We spoke with the doctor.  P2 - We had spoken with the paediatrician, and he referred us to an allergist for confirmation and guidance.  P3 - We had continuous communication with the paediatrician.  P4 - With the doctor’s guidance, everything was fine.  P5 - Yes, the paediatrician and the allergist explained the steps we needed to follow.  P7 - The child has had a milk protein allergy since birth. The diagnosis was made six months ago.  P9 - We concluded it was a milk allergy when the child was 5–6 months old. We followed the paediatrician’s instructions.  P10 - From the paediatrician and the allergist.  P11 - From the paediatrician and the allergist.  P12 - From the paediatrician |
|  |  | Diagnosis by allergist | P2, P3, P4, P5, P6, P7, P8, P10, P11 | P2 - We went to the allergist to perform a skin prick test for confirmation (the test confirmed the allergy).  P3 - The symptoms continued until we went to the allergist.  P4 - We had a skin prick test done by the allergist every six months.  P5 - The paediatrician and the allergist explained the steps we needed to follow.  P6 - We stopped all foods that contained dairy or traces of dairy and went to the allergist.  P7 - The child has had a milk protein allergy since birth. The diagnosis was made six months ago.  P8 - We did a skin prick test with the allergist (the test showed confirmation of the allergy).  P10 - From the paediatrician and the allergist.  P11 - From the paediatrician and the allergist. |
|  |  | Family history | P1, P10 | P1 - My other daughter is allergic to dust, and I remember my brother advised me to always keep one with me because his daughter once had an allergic reaction while playing, and they had to respond immediately.  P10 - I don’t believe she will be psychologically affected, because my cousin’s daughter has a severe allergy to nuts, and overall, I see that she is completely fine with the fact that she must not eat them. |
|  |  | Testing (skin prick test) | P1, P2, P4, P5, P8 | P1 - We went to do the skin prick test, so we came to the allergist’s office.  P2 - We came to do the skin prick test for confirmation (the test confirmed the allergy).  P4 - We had a skin prick test every six months.  P5 - A skin prick test was done to see if the allergy had subsided, but the result remained positive.  P8 - We did a skin prick test (the test confirmed an allergy to legumes). |
|  |  | Hospitalization | P1, P6, P10, P12 | P1 - They stayed there for three nights.  P6 - She was admitted to the hospital, and the doctors could not find what was wrong.  P10 - We stayed there for about two hours until the symptoms passed, then we left and went back in the evening to be checked again. |
|  | Symptoms | First symptoms (e.g., scratching, crying) | P1, P2, P3, P4, P5, P6, P7, P8, P9. P11 | P1 - His face was affected; he was scratching his face and neck, crying, and had watery eyes and a runny nose.  P2, P3, P4, P5 - There was blood in the stool.  P6 - He had diarrhea and blood in the stool.  P7 - Diarrhea, constipation, and eczema.  P8 - The diarrhea did not stop.  P9 - Reflux, and at the beginning we did not know what was causing it.  P11 - He had diarrhea and blood in the stool. |
|  |  | Vomiting | P9 | P9 - When it affects her, she vomits until her stomach is empty. |
|  |  | Body redness | P1, P10 | P1 - When he eats something, he becomes red (especially on his face).  P10 - Her face became red. |
|  |  | Distinguishing mild vs severe symptoms | P1 | P1 - But only here, and I remember doctor told me If the skin turns red it's likely a mild reaction. But if the redness spreads down the neck, throat, and starts to affect breathing it could be more serious. |
|  |  | Eczema | P4, P7 | P4 - He developed many eczema patches on his legs and other areas.  P7 - diarrhoea, constipation, and eczema. |
|  |  | Later symptoms (Immediate reaction to exposure) | P10 | P10 - The next day I gave her egg-and-lemon sauce, and her face became red. |
| **2. Living with FA** | Food Practices (Home) | Cooking at home / avoiding restaurants | P1, P5, P6, P9, P10 | P1 - I cook his food myself. He doesn’t eat food from outside. I don’t go out much - not because of the allergy, but because I prefer staying at home.  P5 - We always prepare food at home that he is allowed to eat.  P6 - I always have things with me; I am prepared.  P9 - We are very careful to bring our own food and do not eat from restaurants; we are fully prepared.  P10 - I am always prepared and take things with me. |
|  |  | Careful introduction of foods | P1, P4 | P1 - Now, whenever I give him something new, I give him a very, very small amount to see if there is a reaction.  P4 - At the first stage, we started introducing baked milk. |
|  |  | Changing milk formula | P3, P9 | P3 - We changed 2–3 types of formula until we found the right one.  P9 – change formulas |
|  |  | Controlled environment at home | P4, P6, P7, P9, P11 | P4 - In front of him, we generally avoid eating anything he cannot have. If he sees it, we tell him, “My love, no, you can’t eat this,” and thankfully he understands.  P6 - We completely avoid all dairy products.  P7 - Anything that contains milk, both for us and for the child.  P9 - Of course, I am very careful not to use any dairy products at all, whether it is for the baby or when I cook for us.  P11 - I always make sure that nothing contains dairy and that substitutes are used instead. |
|  |  | Restricted cooking options | P5, P8 | P5 - It is restrictive.  P8 - We cook a lot of meat; I don’t have many options. |
|  |  | Avoidance (total dairy elimination) | P2, P3, P4, P5, P6, P7, P8, P9, P11 | P2 - We started using hypoallergenic formula.  P3 - I also removed milk from my own diet.  P4 - I avoided all dairy products and traces of dairy.  P5 - he has not eaten any dairy products; hypoallergenic formula.  P7 - We stopped anything that contains milk.  P8 - I started eliminating dairy from my diet.  P11 - I always make sure nothing contains dairy |
|  |  | Hiding allergenic foods | P6 | P6 - Milk protein is everywhere; I once bought a vegan ice cream that contained emulsifiers, and it immediately affected her. |
|  |  | Limited snack options (e.g., egg) | P5, P10 | P5 - We are more limited in what I can cook for him. The food options he can eat are always restricted.  P10 – we have limited options |
|  | Food Practices (Outside) | Sibling food differences | P10 | P10 - My son was eating, and without thinking I asked her if she wanted some too, because she usually wants what we’re eating. Then I realized that the burger contained egg. |
|  |  | Preparing for kindergarten | P1. P6 | P1 - He will be eating homemade food not from the place (kindergarden). |
|  |  | Extended family cooperation | P4, P7, P8 | P4 - The same applies to the close family environment; for example, they will not put cheese on the table.  P7 – we don’t put the foods on the table  P8 - Others also need to learn how to handle it as well (for example, family members and friends). |
|  |  | Restaurants: avoidance and caution | P1, P7, P8, P10 | P1 - I don’t go out because I don’t like it, not because of the allergy. I like to stay at home.  P7 - Yes, we always call the restaurant to see if they have something on the grill without sauce and generally without dairy.  P8 - I need to talk to them so I know what I can eat and what options I have  P10 - I am always prepared and take things with me. |
|  |  | Social awareness (among other parents) | P4, P7 | P4 - I believe there is now enough awareness among parents of young children regarding allergy issues.  P7 - We always call ahead to inform them about the allergy. |
|  |  | Stress from social events | P4, P6, P12 | P4 - There was an incident where he had a severe skin reaction; we gave Xyzal and went to the general hospital. Because it did not happen in front of us, we did not know exactly what caused it. That was very stressful.  P12 - For one year, I was very anxious about his exposure to allergens at kindergarten.  P6 - Situations such as parties or eating outside the home make me quite anxious, because there is never 100% full control. |
|  |  | Preparedness: carrying safe foods | P5, P6, P9 | P5 - We always prepare food at home that he can eat, because you never know what will be available (whether they will actually have something without dairy).  P6 - I always have things with me; I am prepared.  P9 - We are careful not to use any dairy products at all, whether it is for the baby or when I cook something for ourselves.  P10 - I am always prepared and take things with me. |
|  |  | Constant daily preparation routines | P5, P9 | P5 - We always prepare in advance.  P9 - We bring our own food and do not eat food from restaurants |
|  | Psychological and Emotional | Parental anxiety | P1, P3, P4, P5, P6, P7, P8, P10, P11, P12 | P1 - Every time he eats something, I feel like I might pass out.  P3 - I feel anxious, but I try to manage it and not show it.  P4 - We had some anxiety during the food introduction stage.  P5 - It is stressful, and I always have some level of worry.  P6 - A lot of anxiety, because the risk of accidentally consuming food with traces of milk protein is high.  P7 - Anxiety. It is difficult and it certainly has an impact.  P8 - At the beginning, when he had constant diarrhea or was not gaining weight, it caused me anxiety.  P10 - I worry about the fact that she cannot eat something that I might cook  P12 - For one year, I was very anxious about his exposure to allergens at kindergarten. |
|  |  | Husband as calm presence | P1 | P1 - Thank God my husband is very, I am very emotional, he is very calm down, so this is helping me a lot. |
|  |  | Trust in doctors | P2, P5, P6, P9 | P2 - We will follow the doctor’s instructions, whatever he tells us.  P5 - I assume that in such cases we will have guidance from the doctor on how to manage it.  P6 - We try to stay strong and follow the allergist’s advice.  P9 - We followed the pediatrician’s instructions and did not have any issues. |
|  |  | Advice for parents | P1 | P1 - The first thing what parents have to know how to do is to calm down. The first thing, calm down, because when you are stressed, you lose the game. You cannot take a decision |
|  |  | Relationship with doctors | P1 | P1 - have a very good relationship with the doctor – to feel confident to contact him |
|  |  | Hope for the future | P3, P5 | P3 - I hope everything will be okay and that she will not struggle because of the allergy.  P5 - I want to believe that everything will go well and that she will overcome it soon. |
|  |  | Parental sadness (child missing out) | P4 | P4 - It makes me sad if my child is deprived of something he could otherwise try in foods and enjoy a meal the way we do. |
|  |  | Child’s frustration and sadness | P6, P7, P8, P9 | P6 - There are times when she asks for things and I have to tell her “no,” and this creates tension-she gets angry and upset.  P7 - She has already started noticing and wanting things. For example, treats are given at school, and I feel that this affects her.  P8 - Everything is affected-the whole household is affected.  P9 - From infancy, around 2–3 months old, she would see a bottle and cry; she is afraid that it will cause a reaction. |
|  |  | Child aware of restrictions | P4, P7 | P4 - We tell him, “My love, no, you can’t eat this,” and thankfully he understands.  P7 - Now he has started to understand that he cannot eat certain things and asks about them. |
|  |  | Exposure to food at school | P6, P9 | P6 - The risk of accidentally consuming food with traces of milk protein is high.  P9 - consuming food with milk is very high. |
| **3. FA Management** | Medical Management | Antihistamine (Xyzal) | P1, P3, P4, P6, P10 | P1 - So we gave him Xyzal (an antihistamine).  P3 - We spoke with the doctor and gave him Xyzal.  P4 - We always have Xyzal in our bag.  P6 - We always carry antihistamines with us.  P10 - I called the pediatrician immediately, and he told me to give Xyzal. |
|  |  | Avoidance | P1, P3, P4, P5, P6, P7, P8, P9 | P1 - We need to know exactly what he is allergic to and inform others that it is dangerous for him and that no one should give it to him.  P3 - Yes, we replaced dairy products in our diet with almond and soy  P4 - We avoid eating anything that he cannot eat.  P5 - So far, he has not eaten any dairy products.  P6 - We completely avoid all dairy products.  P7 - Anything that contains milk, both for us and for the child.  P8 - I removed eggs and dairy from my diet, believing that I should not eat anything that might affect the baby.  P9 - I make sure not to use any dairy products at all. |
|  |  | Adrenaline injection (EpiPen - Yes) | P1, P10 | P1 - The doctor also told us about it, and we ordered an EpiPen.  P10 - Yes, I know how to use it, but the idea to use it makes me anxious. |
|  |  | Adrenaline injection (EpiPen - No) | P3, P4, P5, P6, P7, P8, P9 | P3 - I am informed about how to use it, and I feel that we will not need it.  P4 - No, we only have Xyzal.P5 – όχι  P6 - We were not informed about the EpiPen.  P7 - We were not informed about the EpiPen.  P8 - No, I don’t know anything about it.  P9 - We did not receive any information at all. |
|  |  | Hospital admission/intravenous medicines | P1, P4, P12 | P1 - They were giving him intravenous medication.  P4 - There was an incident where he had a severe skin reaction; we gave Xyzal and went to the general hospital.  P12 - We went to the emergency department, and he stayed at Makario Hospital for three nights. |
|  |  | Follow-up examinations | P1, P4 | P1 - I would take him to the doctor a hundred times until I know he is okay.  P4 - We had a skin prick test every six months. |
|  |  | Importance of carrying EpiPen | P1 | P1 - It’s important to carry an EpiPen for emergencies |
|  |  | Stopping breastfeeding/maternal dietary changes | P3, P8 | P3 - We had continuous communication with the paediatrician, and he also told me to stop breastfeeding for a while.  P8 - First, I removed dairy from my diet and then, after one month, eggs. When I removed dairy, the symptoms stayed the same, but after removing eggs there was a noticeable improvement. However, with legumes there are still symptoms, such as diarrhoea and vomiting. |
|  |  | Trying multiple formulas | P3, P9 | P3 - We changed 2–3 types of formula until we found the right one.  P9 - It took several months until we settled on a formula that did not cause her any discomfort. |
|  |  | Accidental exposure | P1, P3, P6, P8, P10 | P1 - Sometimes, when he eats something, he becomes red, especially on his face.  P3 - His grandfather once tried to give him cream with milk, and he had a reaction again.  P6 - There was an occasion when she ate something and shortly afterward experienced severe pain.  P10 - Yes, there was a time when I gave her a homemade burger that I had prepared. |
|  |  | Repeated skin prick tests | P4, P8 | P4 - We had a skin prick test every six months.  P8 - We did a skin prick test for the second time, and the test confirmed an allergy to legumes. |
|  | Preparedness | Gradual introduction of baked milk/cheese | P4 | P4 - At the first stage, we started introducing baked milk. |
|  |  | Always carrying medication | P1, P4, P6, P7 | P1 - I immediately give him Xyzal.  P4 - We always have Xyzal in our bag.  P6 - We always carry antihistamines with us.  P7 - I carry Xyzal with me. |
|  |  | Keeping food-symptom diaries | P8 | P8 - I kept a diary and recorded the symptoms from the time I started eliminating foods. |
| **4. Educational and Societal Awareness** | Need for Educational Resources | Practical guides | P1, P3, P5, P6, P10, P11 | P1 - Yes, because then parents can be prepared in advance. They can learn what to do in such situations, because if you haven’t experienced it before, you don’t know how to respond. It’s like cutting your hand, you know how to put on a bandage. The steps are clear.  P3 - There should be proper information, in the form of an informational leaflet.  P4 - Proper information in general about when food introduction should take place.  P5 - More guidance on correct management, alternatives, and what the appropriate action is.  P6 - There should be proper information and education.  P10 - Perhaps clear guidance on which steps need to be followed.  P11 - Proper overall information about the steps that should be followed. |
|  |  | Digital materials | P2, P4, P12 | P2 - In the form of a leaflet or in electronic format.  P4 - electronic format, because that way you always have it with you.  P12 - A mobile app. |
|  |  | Guidance on symptoms | P1, P3 | P1 - Parents can be prepared in advance and learn what to do  P3 - They know how to manage it.  P7 - Explanation of symptoms and guidance, since it is difficult at the beginning. |
|  |  | Allergen-specific food lists | P2, P5, | P2 - It should be clearly stated, depending on the allergy, which foods can and cannot be consumed.  P5 - Based on each baby’s allergy, there should be proper guidance on what would be appropriate for them to eat. |
|  | Schools Awareness | Awareness in kindergartens | P3, P6, P7 | P3 - There is proper information provided in kindergartens.  P6 - A printed leaflet that could be given to relatives or to the school for information.  P7 - A list of foods so that you know what causes reactions and what the child can consume. |
|  | Doctor Awareness | Pediatric guidance gaps | P4, P6, P7, P9 | P4 - I understand that the internet exists, but we always wanted to rely on doctors’ guidance. There is a gap there.  P6 - Overall, better guidance is needed from paediatricians.  P7 – Better guidance from paediatricians  P9 – There should be proper information provided by paediatricians. |
|  | Restaurant Awareness | Restaurant allergen labelling gaps | P8, P10 | P8 - Products should clearly indicate allergens - for example, restaurant menus should be more detailed.  P10 - Restaurants or hotels should be clear and transparent, so you know which foods contain allergens. |

Note: The quotes above were translated from Greek into English.
