# Supplementary material for: Quality of life in families of infants and toddlers with food allergies: A mixed‐methods study
Source: Pediatr Allergy Immunol. 2026 Aug 13;37(8):e70453. doi: 10.1111/pai.70453 (PMC13473807; doi:10.1111/pai.70453)
Supplement: Supplementary file 3 [file PAI-37-e70453-s003.docx]

**Supplemental Table S4.** Demographics of mother and children with FA

| Participant | Maternal Age | Residence | Region | Child age | Age of Diagnosis (consultant allergist or consultant paediatrician) | Child Gender | Child Allergy |
| --- | --- | --- | --- | --- | --- | --- | --- |
| 1 | 35 | Limassol | City | 2-year-old | 16 months | Boy | Tree Nuts |
| 2 | 39 | Nicosia | City | 4 months | 4 months | Boy | Milk protein |
| 3 | 29 | Nicosia | City | 1.5 years old | 1.5–2 years old | Girl | Milk protein |
| 4 | 36 | Nicosia | City | 3 years old | 3.5–4 months | Boy | Milk protein |
| 5 | 26 | Limassol | City | 10 months | Soon after birth (~few days old) | Boy | Milk protein |
| 6 | N/A | Nicosia | N/A | 3 years old | 12 months | Girl | Milk protein |
| 7 | N/A | Nicosia | N/A | 2 years old | Diagnosed: 4 months, confirmed later | Girl | Milk protein |
| 8 | 34 | Limassol | City | 1 year old | ~6–12 months | Girl | Milk protein |
| 9 | N/A | Nicosia | N/A | 1.5-year-old | 5–6 months | Girl | Milk protein |
| 10 | 26 | Limassol | City | 1 year old | 12 months | Girl | Egg |
| 11 | 32 | Limassol | City | 10 months | 3 months | Boy | Milk protein |
| 12 | 38 | Nicosia | City | 2 years old | 11 months | Boy | Egg |
