# Supplementary material for: Quality of life in families of infants and toddlers with food allergies: A mixed‐methods study
Source: Pediatr Allergy Immunol. 2026 Aug 13;37(8):e70453. doi: 10.1111/pai.70453 (PMC13473807; doi:10.1111/pai.70453)
Supplement: Supplementary file 4 [file PAI-37-e70453-s004.docx]

**Supplementary Table S1:** Good Reporting of A Mixed Methods Study (GRAMMS) checklist

| **GRAMMS Item** | **Reported on** |
| --- | --- |
| 1. Describe the justification for using a mixed-methods approach to the research question | Section 1. Introduction, p. 3-4. The study justifies a sequential mixed-methods design to combine quantitative QoL measurement with qualitative exploration of lived caregiving experience. |
| 2. Describe the design in terms of the purpose, priority, and sequence of methods | Section 2. Methods, Section 2.5 (Data Analysis), p. 7–8 (paragraph describing sequential explanatory mixed-methods design and joint display integration). |
| 3. Describe each method in terms of sampling, data collection, and analysis | Section 2. Methods, Quantitative: Section 2.1 (Participants, p. 4–5); Section 2.2 (FAQLQ-PF, p. 5–6); Section 2.5.1 (Quantitative Analysis, p. 7). Qualitative: Section 2.4 (Semi-Structured Interviews, p. 6–7); Section 2.5.2 (Qualitative Analysis and Integration, p. 7–8). |
| 4. Describe where integration has occurred, how it has occurred, and who has participated in it | Integration described in Methods, Section 2.5.2 (p. 7-8) and presented in Results, Section 3.3 (pp. 22–23) with joint display (Table 6). Integration was conducted by EP and SAN, with input from all authors. |
| 5. Describe any limitation of one method associated with the presence of the other method | Discussion, Section 4 (pp. 26–28). Methodological limitations and interaction between methods are acknowledged. |
| 6. Describe any insights gained from mixing or integrating methods | Results, Section 3.3 (pp. 22–25) and Discussion (pp. 26–27) |

**Reference:** O'Cathain A, Murphy E, Nicholl J. The quality of mixed methods studies in health services research. J Health Serv Res Policy. 2008;13(2):92-98.
